# Supplementary material for: Integrated analysis of the aqueous humor microbiome and lens capsule transcriptome in high myopia cataract: a pilot study
Source: Front Med (Lausanne). 2026 Jun 16;13:1845205. doi: 10.3389/fmed.2026.1845205 (PMC13314463; doi:10.3389/fmed.2026.1845205)
Supplement: Supplementary file 3 [file Data_Sheet_1.zip › 4.Meta_Statistic/C372089/Wilcoxon/Family/Family.Wilcoxon.diff.heatmap.pdf]

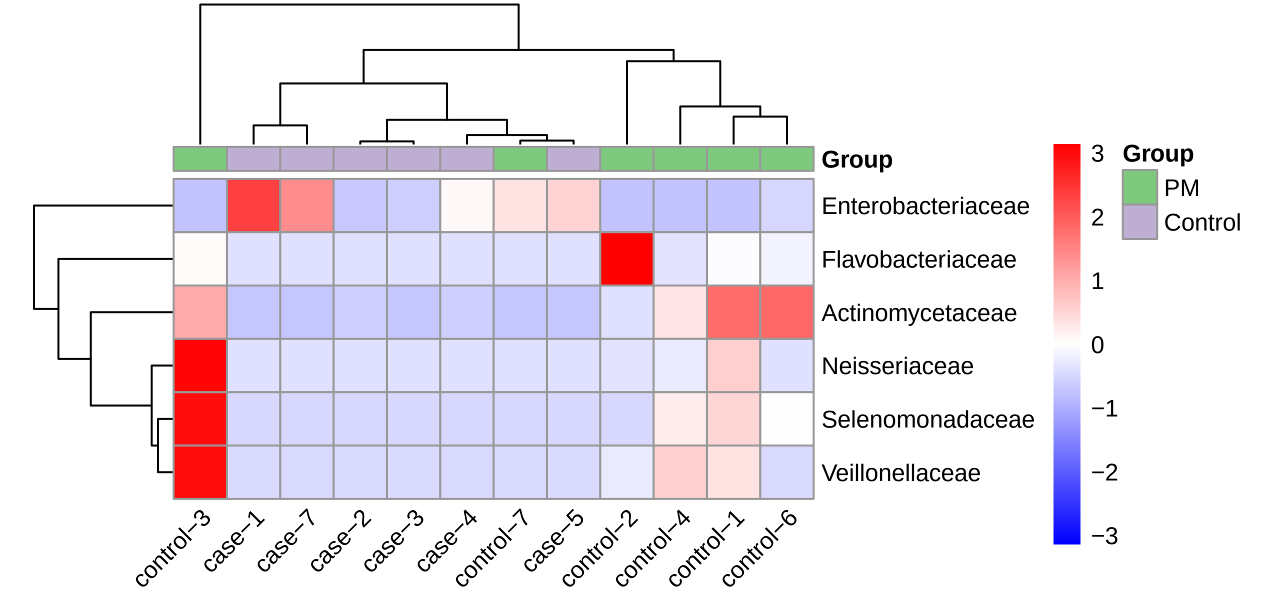

Group
Enterobacteriaceae
Flavobacteriaceae
Actinomycetaceae
Neisseriaceae
Selenomonadaceae
Veillonellaceae
control−3
case−1
case−7
case−2
case−3
case−4
control−7
case−5
control−2
control−4
control−1
control−6
3
2
1
0
−1
−2
−3
Group
PM
Control
